# Safety of Percutaneous Femoral Access for Endovascular Aortic Aneurysm Repair Through Previously Surgically Exposed or Repaired Femoral Arteries

**DOI:** 10.1177/15266028221092980

**Published:** 2022-05-06

**Authors:** Max M. Meertens, Emanuel R. Tenorio, Charlotte C. Lemmens, Giulianna B. Marcondes, Guilherme B. B. Lima, Geert Willem H. Schurink, Bernardo C. Mendes, Gustavo S. Oderich, Barend M. E. Mees

**Affiliations:** 1 Department of Vascular Surgery, Maastricht University Medical Center, Maastricht, The Netherlands; 2 European Vascular Center Aachen-Maastricht, Aachen, Germany; 3 Division of Vascular and Endovascular Surgery, University of Texas Health Science, Houston, TX, USA; 4 Division of Vascular and Endovascular Surgery, Mayo Clinic, Rochester, MN, USA

**Keywords:** percutaneous access, transfemoral access, endovascular aortic repair, technical success, access complications

## Abstract

**Objective:**

Percutaneous femoral artery access is being increasingly used in endovascular aortic repair (EVAR). The technique can be challenging in patients with previously surgically exposed or repaired femoral arteries because of excessive scar tissue. However, a successful percutaneous approach may cause less morbidity than a “re-do” open femoral approach. The aim of this study was to assess the impact of prior open surgical femoral exposure on technical success and clinical outcomes of percutaneous approach.

**Methods:**

This study retrospectively reviewed the clinical data of patients who underwent percutaneous EVAR between 2010 and 2020 at 2 major aortic centers. Patients were divided into 2 groups (with or without prior open surgical femoral access) for analysis of clinical outcomes. Only punctures with sheaths ≥12Fr were included for analysis. The access and (pre)closure techniques were similar in both institutions. Primary end points were intraoperative technical success, access-related revision, and access complications. A multivariate analysis was performed to identify determinants of conversion to open approach and femoral access complications in intact and re-do groins.

**Results:**

A total of 632 patients underwent percutaneous (complex) EVAR: 98 had prior open surgical femoral access and 534 patients underwent de novo femoral percutaneous access. A total of 1099 femoral artery punctures were performed: 149 in re-do and 950 in intact groins. The extent of endovascular repair included 159 infrarenal, 82 thoracic, 368 fenestrated/branched, and 23 iliac branch devices. No significant differences were seen in technical success (re-do 93.3% vs intact 95.3%, p=0.311), access-related surgical revision (0.7% vs 0.6%, p=0.950), and access complications (2.7% vs 4.0%, p=0.443). For the whole group, significant predictors for access complications in multivariate analyses were main access site (odds ratio [OR] 2.39; 95% confidence interval [CI] 1.07%–5.35%; p=0.033) and increase of the procedure time per hour (OR 1.65; 95% CI 1.34%–2.04%; p<0.001), while increase in sheath-vessel ratio had a protective effect (OR 0.33; 95% CI 0.127%–0.85%; p=0.021). Surgical conversion was predicted by main access site (OR 2.32; 95% CI 1.28%–4.19%; p=0.007) and calcification of 50% to 75% of the circumference of the access vessel (OR 3.29; 95% CI 1.38%–7.86%; p=0.005).

**Conclusion:**

Within our population prior open surgical femoral artery exposure or repair had no negative impact on the technical success and clinical outcomes of percutaneous (complex) endovascular aortic aneurysm repair.

## Introduction

The importance of endovascular aortic repair (EVAR) has increased and become a relevant alternative to a surgical approach in patients with aortic aneurysms, including those with more complex aortic aneurysms.^1–3^ Arterial access during EVAR and complex EVAR was traditionally obtained using open surgical exposure of the common femoral artery.^4^ However, percutaneous transfemoral access provides a safe and effective alternative arterial access strategy. In 2017, a meta-analysis showed that percutaneous endovascular aortic repair (pEVAR) and the usage of arteriotomy closure devices reduced the incidence of postoperative seromas, wound dehiscence, and surgical site infections.^5^ Furthermore, percutaneous transfemoral access provides periprocedural advantages, such as shorter time to hemostasis and procedure completion and favorable trends in blood loss, groin pain, and overall quality of life.^6^

Because of increased endovascular treatment options patients often undergo more than 1 transfemoral endovascular procedure. Especially in patients who had a prior surgical exposure or repair of the femoral artery in the context of an endovascular procedure or due to prior revascularization the access strategy has to be evaluated carefully. Re-do open surgical access of the femoral arteries is time consuming and technically more challenging than primary femoral artery exposure. However, percutaneous transfemoral access using larger profile devices is often avoided because of a perceived high rate of failures or increased risk of complications.^7,8^ The risk of access-related revisions is described to be significantly higher in the presence of groin fibrosis.^7^ Large-bore hole punctures are known to be more challenging and are responsible for most access complications in endovascular procedures.^6,9,10^

The aims of this study were to assess the impact of previous surgical femoral artery exposure or repair on the technical success and postoperative clinical outcomes of percutaneous access for EVAR, as well as to identify determinants of conversion to open approach and access complications.

## Methods

### Study Design

The study was a retrospective review of patients who underwent pEVAR at 2 major aortic centers between 2010 and 2020. It was approved by the institutional review board of both centers. Due to the retrospective character of the study, informed consent was not required.

Patients who underwent EVAR with no prior femoral access (intact) or with prior surgical femoral artery exposure or repair (re-do) from 1 or both groins were included. We excluded patients treated with prior percutaneous transfemoral access from the analysis. Furthermore, we excluded groins in which the largest introduced sheath was <12Fr or unknown. For each patient, both groins were evaluated separately. When a patient had 1 re-do and 1 intact groin, the patient was registered as a re-do patient and the intact groin was excluded.

### Data Collection

Hospital records were reviewed retrospectively to collect patient, groin, and procedural characteristics by screening the digital patient dossiers and preprocedural computed tomography (CT) scans. Patient characteristics included demographics, comorbidities, and preoperative use of antithrombotic medication. Groin characteristics included previous surgical or percutaneous access through the groin, vessel diameter, femoral artery calcification, and groin scar visible on CT scan. Procedural characteristics were type of EVAR, percutaneous access site, main access site, maximum sheath size, and the sheath-vessel ratio. The sheath-vessel ratio for each groin was calculated by dividing the vessel diameter through the maximal sheath size. The main access site was defined as the access site through which the largest sheath was introduced.

### End Points

As we hypothesized that percutaneous access in re-do groins would be equally safe as in intact groins, our primary end point focused on the technical success of percutaneous femoral access in large-bore hole punctures. Technical success was defined as the absence of intraoperative conversion to surgical femoral artery access. The secondary end points were the safety outcomes of percutaneous access, mortality, and general complications within 30 days. Safety outcomes were access complications, such as lower limb ischemia, groin hematoma, peripheral neuropathy, femoral artery dissection, bleeding, pseudoaneurysm, and the need for postoperative access-related surgical revision within 30 days. A composite end point was created by including both postoperative access-related surgical revisions and intraoperative conversions and defined as any surgical femoral artery revision. General complications were defined as myocardial infarction, acute heart failure, respiratory failure, pneumonia, stroke/transient ischemic attack, spinal cord injury, acute kidney injury, and gastrointestinal complications.

### Selection Criteria for Percutaneous Access

The applied selection criteria were equal for intact and re-do groins. A preprocedural CT scan was performed from each patient to plan the procedure including the access strategy. Criteria evaluated before choosing percutaneous access were: calcification, diameter, and stenosis of the femoral and external iliac artery. A percutaneous access was chosen if the access vessel had a diameter >6 mm and was free from anterior calcification. The common femoral artery was the preferential access site, but in case of a high bifurcation the superficial femoral artery was used. The access technique was chosen by the senior operating physician at each institution. All physicians were experienced in the use of ProGlide (Abbott Vascular, Santa Clara, Calif) and Angioseal (St Jude Medical, Minnetonka, Minnesota) devices.

### Percutaneous Access Technique

All punctures were performed using ultrasound guidance. In the presence of stenosis or severe calcification, the puncture was performed proximally from the lesion. In patients with previously surgically exposed or repaired femoral arteries, the puncture was performed using a micropuncture set or a 4Fr dilator. Subsequently, the access was progressively dilated over a short stiff guidewire (75-cm Amplatz, Boston Scientific, Marlborough, Massachusetts) before introducing the ProGlide. In both institutions, closure of all large-bore holes (>10Fr) was performed by applying a preclose technique with 2 ProGlides.

### Closure Technique

Both institutions followed the same closure protocol by tightening the ProGlides according to instructions for use, including 5 minutes of manual compression for minor residual bleeding. In case of significant persistent bleeding, an additional ProGlide or Angioseal was placed. When this was not sufficient for adequate hemostasis, additional manual compression was applied for 15 minutes before conversion, unless it was already apparent to the surgeon that compression would not induce hemostasis. If patients received a conversion for persistent bleeding, this was characterized as “conversion for unsuccessful hemostasis.” Patients did not receive protamine routinely, but only in case of significant persistent bleeding needing manual compression after placement of an additional closure device.

### Statistics

The 2 groups were compared using Pearson’s chi-square or Fisher’s exact test for analysis of categorical variables. Continuous variables were analyzed by using Student’s *t*-test. Predictors of conversion to surgical approach and access complications were sought separately using univariate and multivariate regression analyses. The results were presented as the odds ratio (OR), with 95% confidence intervals (CIs). Multivariate testing was conducted by including all potential covariates (p<0.1) from the univariate analyses into a backwards stepwise logistic regression model. Categorical variables are presented as percentages and continuous variables as mean and standard deviation. The significance level was set for p-values <0.05. Data were analyzed using SPSS Version 25.0 (IBM, New York, NY).

## Results

### Patient and Groin Characteristics

We included a total of 632 patients treated with (complex) EVAR, 256 from the Maastricht University Medical Center, the Netherlands, and 376 from the Mayo Clinics Rochester, USA. Out of 632 patients, 98 received percutaneous access after previous surgical exposure or repair of the femoral arteries and 534 patients had de novo femoral percutaneous access. In total, 1099 femoral arteries were approached with sheaths ≥12Fr through 149 re-do and 950 de novo groins. Patients with prior surgical access of the femoral artery more often had peripheral arterial disease (PAD; 34.7% vs 17.3%, p<0.001) and hypertension (84.7% vs 73.7%, p=0.021). Furthermore, the patients in the re-do groups were more often classified as American Society of Anesthesiologists (ASA) III and the de novo group as ASA II. There were no significant differences in the preoperative anticoagulation regime between both groups. Five patients (5.1%) had a prior endarterectomy of the femoral artery for PAD, 4 (4.1%) a prior femoral-femoral bypass graft, 3 patients (3.1%) underwent open aortic aneurysm repair including exposure of the common femoral artery, 1 patient (1%) received an aortofemoral bypass, and 1 patient (1%) had undergone an EVAR with endarterectomy of the femoral artery. The remaining 84 patients (85.7%) had a previous oblique surgical approach and primary closure of the femoral artery during an endovascular procedure. The performed procedures were EVARs (20.5% vs 23.6%, p=0.419), thoracic EVARs (12.2% vs 13.1%, p=0.815), fenestrated and branched EVARs (FB-EVARs, 55.1% vs 58.8%, p=0.495), Chimney-EVARs (3.1% vs 1.9%, p=0.459), and iliac branch device (IBD, 9.2% vs 3.2%, p=0.34).

The demographics, preoperative anticoagulation regime, and performed interventions are summarized in Table 1.

**Table 1.** Patient Characteristics and Performed Procedures.

	Re-do femoral access (n=98 patients)	De novo femoral access (n=534 patients)	p value
Male gender	82 (83.7)	421 (78.8)	0.275
Age, years (SD)	73.5 (7.9)	72.3 (9.9)	0.252
BMI, kg/m^2^ (SD)	27.1 (6.0)	27.4 (4.9)	0.658
Height, cm (SD)	175.6 (8.9)	174.5 (8.7)	0.178
Weight, kg (SD)	82.7 (16.5)	83.1 (17)	0.854
Current smoker	33 (34.4)	131 (24.7)	0.046
Former smoker	39 (40.2)	239 (45)	0.381
Coronary artery disease	42 (42.9)	190 (35.6)	0.175
Chronic heart failure	9 (9.3)	44 (8.3)	0.738
Dialysis	2 (2%)	3 (0.6)	0.174
COPD	24 (24.5)	114 (21.5)	0.507
Hypertension	83 (84.7)	393 (73.7)	0.021
Dyslipidemia	72 (73.5)	349 (65.5)	0.123
Previous stroke/TIA	14 (14.3)	77 (14.4)	0.967
Peripheral arterial disease	34 (34.7)	92 (17.3)	<0.001
Diabetes mellitus	14 (14.3)	66 (12.4)	0.608
ASA			
1	0	4 (0.7)	1
2	33 (33.7)	244 (45.7)	0.028
3	57 (58.2)	235 (44)	0.010
4	7 (7.1)	46 (8.6)	0.629
Acetylsalicylic acid	66 (67.3)	305 (57.2)	0.061
Clopidogrel	14 (14.3)	73 (13.7)	0.876
Other platelet inhibitor	3 (3.1)	5 (0.9)	1
Dual antiplatelet therapy	4 (4.1)	26 (4.9)	1
DOAC	6 (6.1)	36 (6.8)	0.814
Vitamin K antagonist	11 (11.3)	53 (10)	0.680
Statin	48 (49)	252 (47.3)	0.757
Performed procedure			
EVAR	20 (20.5)	127 (23.6)	0.419
TEVAR	12 (12.2)	70 (13.1)	0.815
F/B EVAR	54 (55.1)	314 (58.8)	0.495
Chimney-EVAR	3 (3.1)	9 (1.7)	0.359
IBD	9 (9.2%)	14 (3.2)	0.004

Values in parentheses are percentages unless indicated otherwise.

Abbreviations: ASA classification, American Society of Anesthesiologists classification; BMI, body mass index; COPD, chronic obstructive pulmonary disease; DOAC, direct oral anticoagulation; EVAR, endovascular aortic repair; F/B EVAR, fenestrated/branched endovascular aortic repair; IBD, iliac branch device; SD, standard deviation; TEVAR, thoracic endovascular aortic repair; TIA, transient ischemic attack.

### Procedure and Access Characteristics

In the re-do group, the right femoral artery was accessed 82 times and the left 67 times. In the de novo group, the right femoral artery was percutaneously accessed 514 times and the left femoral artery 436 times. In 68 (45.6%) of the prior surgically exposed groins, scar tissue was identifiable on the preoperative CT scan. The femoral artery diameter was larger in patients with prior surgical femoral artery exposure (9.7 vs 9.2 mm, p=0.004). Overall, there were no significant differences in the calcification pattern. The maximal sheath size of the largest sheath was equal between both groups (18.7 vs 18.8Fr, p=0.697). Grouping the used sheath sizes from 12 to 16 (23.5% vs 25%, p=0.415), 17 to 20 (57.7% vs 54.9%, p=0.326), and larger than 20Fr (18.8% vs 20.1%, p=0.443), we found no significant difference between both groups. Standard closure after large sheath punctures with 2 ProGlides was performed in 129 (86.6%) re-do groins versus 856 (90.1%) intact groins (p=0.256). Also, there were no significant differences in the usage of additional ProGlides (6% vs 5.5%, p=0.783). In 20 (13.4%) re-do groins versus in 94 (9.9%) intact groins, it was necessary to insert an additional Angioseal after closure with 2 ProGlides (p=0.189). The groin and access characteristics are summarized in Table 2.

**Table 2.** Procedure and Access Characteristics.

	Re-do femoral access (n=149 groins)	De novo femoral access (n=950 groins)	p value
Diameter, mm (SD)	9.7 (2.5)	9.2 (2.2)	0.005
CFA calcification			
Lateral	25 (21.9)	161 (20.4)	0.697
Anterior	11 (10)	45 (6.1)	0.123
Posterior	57 (46.3)	320 (40.3)	0.205
Percentage of the circumference			
0%–25%	72 (48.3)	530 (55.8%)	0.089
25%–50%	57 (38.3)	333 (35.1)	0.448
50%–75%	6 (4%)	38 (4)	0.988
75%–100%	8 (5.4)	39 (4.1)	0.478
Groin scar on CT scan	68 (45.6)	0	<0.001
Percutaneous access site			
Left	67 (45.9)	436 (514)	
Right	82 (55)	514 (54.1)	0.833
Main access site	85 (57)	504 (59.6)	
Secondary access site	64 (43)	446 (40.4)	0.378
Sheath size diameter			
12–16Fr	35 (23.5)	238 (25)	0.415
17–20Fr	86 (57.7)	521 (54.9)	0.326
>20Fr	28 (18.8)	191 (20.1)	0.443
Mean sheath size	18.7	18.8	0.697
Sheath-vessel diameter ratio (SD)	1.60 (0.46)	1.48 (0.46)	0.006
Closure device			
ProGlide only	121 (81.2)	810 (85.3)	0.256
Additional ProGlide	8 (5.4)	46 (4.8)	0.783
Additional Angioseal	20 (13.4)	94 (9.9)	0.189

Values in parentheses are percentages unless indicated otherwise.

Abbreviations: CFA, common femoral artery; CT, computed tomography; Fr, French gauge; SD, standard deviation.

### Thirty Day Clinical Outcomes

There were no significant differences between both groups in mortality (30 day and in-hospital) or general complications, such as myocardial infarction, acute heart failure, respiratory failure, pneumonia, cerebrovascular events, acute kidney injury, and gastrointestinal complications, or mean hospital stay. The general outcomes are summarized in Table 3.

**Table 3.** Patient Outcomes.

	Re-do femoral access (n=98 patients)	De novo femoral access (n=534 patients)	p value
Time to discharge (days)	5.4	6.2	0.484
Death	1 (1.1)	18 (3.4)	0.497
Myocardial infarction	1 (1.1)	5 (0.9)	0.854
Acute heart failure	1 (1.1)	7 (1.3)	1
Respiratory failure	3 (3.3)	12 (2.2)	0.405
Pneumonia	4 (4.4)	16 (3.0)	0.505
Stroke	2 (2.2)	8 (1.5)	0.637
TIA	0	5 (0.9)	1
Spinal cord injury	3 (3.3)	10 (1.9)	0.447
Acute kidney injury	6 (6.6)	40 (7.5)	0.850
Gastrointestinal complications	0	16 (3.0)	0.146

Values in parentheses are percentages unless indicated otherwise.

Abbreviation: TIA, transient ischemic attack.

### Primary and Secondary End Points

The technical success of percutaneous femoral access was 93.3% in the re-do group, which did not significantly differ from 95.3% in the intact groin group (138 vs 905 groins, p=0.311).

In the re-do group, 10 access-related conversions (6.7%) had to be performed. Four were needed because of the inability to deploy the ProGlides and to perform the preclose technique, 3 due to an inability to introduce the sheath at the beginning of the procedure, and 2 conversions were done at the end of the procedure because of unsuccessful hemostasis. Nine conversions were needed in groins which were prior exposed with oblique incision for femoral access in prior endovascular procedures. One conversion was required for bleeding from the access puncture after dislocation of the 16-Fr sheath, in a groin which was previously exposed for an endarterectomy of the common femoral artery.

In the de novo group, 45 conversions were needed. Eight were performed at the start of the procedure, because of failure to introduce or deploy the ProGlides (n=7) or inability to introduce the sheath (n=1). During the procedure, 8 conversions were required, due to external iliac artery rupture (n=3), due to bleeding of the common femoral artery (n=4), or due to a dissection of the common iliac artery (n=1). At the end of the procedure, another 29 conversions had to be performed because of unsuccessful hemostasis (n=19), ischemia after closure (n=8), or thromboembolic complications (n=2).

No significant differences between the 2 groups were seen regarding overall access complications (2.7% vs 4.0%, p=0.443), neither when comparing different access complications separately (lower limb ischemia, groin hematoma, peripheral neuropathy, femoral artery dissection, bleeding, pseudoaneurysm). The need for postoperative surgical revision of an access complication within 30 days did also not differ significantly between groups (1 [0.7%] vs 6 [0.6%], p=0.95). Finally, the combined end point of any surgical femoral revision did also not differ between both groups (11 [7.4%] vs 51 [4.6%], p=0.312; Table 4).

**Table 4.** Primary and Secondary Outcomes.

	Re-do femoral access (n=148)	De novo femoral access (n=950)	p value
Technical success	138 (93.3)	905 (95.3)	0.311
Access complications	4 (2.7)	38 (4.0)	0.443
Hematoma	1 (0.7)	13 (1.4)	0.483
Neuropathy	1 (0.7)	2 (0.2)	0.280
Dissection	0	12 (1.3)	0.258
Active bleeding	1 (0.7)	7 (0.7)	0.931
Pseudoaneurysm	0	3 (0.3)	1
Ischemia	1 (0.7)	8 (0.8)	0.404
Wound infection	0	3 (0.3)	1
Surgical revision	1 (0.7)	6 (0.6)	0.950
Any surgical femoral artery revision	11 (7.4)	51 (5.4)	0.312

Values in parentheses are percentages.

### Univariate and Multivariate Analyses for Determinants of Surgical Conversion and Access Complications

For these analyses, 1098 punctures with a sheath ≥12Fr were included. Fifty-eight (5.2%) groins needed intraoperative surgical conversion and 42 groins (3.8%) had access complications. In the univariate analysis, calcification of 50% to 75% of the circumference of the common femoral artery (OR 3.72; 95% CI 1.57–8.76; p=0.003), main device access site (OR 2.371, 95% CI 1.32–4.27; p=0.004), and IBD procedure (OR 2.882, 95% CI 1.08–7.68; p=0.032) were independent risk factors for surgical conversion. A multivariate analysis confirmed that calcification of 50% to 75% of the circumference of the common femoral artery (OR 3.29; 95% CI 1.38–7.86; p=0.005) and main device access site (OR 2.32; 95% CI 1.28–4.19; p=0.007) increased the risk of surgical conversion.

History of PAD (OR 2.28; 95% CI 1.18–4.42; p=0.014), increase per Fr sheath size (OR 1.25; CI 1.09–1.44; p=0.002), increase of the procedure time per hour (OR 1.65; CI 1.34–1.96; p<0.001), and main device access site (OR 2.52; 95% CI 1.25–5.07; p=0.01) were significant independent risk factors for the development of access complications in univariate analysis. A multivariate analysis confirmed that main device access site (OR 2.39; 95% CI 1.07–5.35; p=0.033) and increase of the procedure time per hour (OR 1.65; CI 1.34–2.04; p <0.001) were relevant risk factors for access complications, while an increase in sheath-vessel ratio had a protective effect (OR 0.33; CI 0.13–0.85; p=0.021). The univariate and multivariate analyses are summarized in Tables 5 and 6.

**Table 5.** Univariate and Multivariate Regression Analysis for Determinants of Conversion to Open Approach After Large Sheath Size Punctures.

	Odds ratio	95% CI lower limit	95% CI upper limit	p value
Univariate				
Prior surgical access	1.352	0.668	2.734	0.401
Male gender	1.118	0.581	2.149	0.739
Diabetes mellitus	1.127	0.522	2.434	0.761
Previous stroke	1.333	0.658	2.699	0.542
Increase per Fr sheath size	1.104	0.993	1.227	0.066
Calcification 50% to 75% of the CFA circumference	3.724	1.583	8.761	0.003
IBD	2.882	1.081	7.680	0.032
Main device access site	2.371	1.316	4.270	0.004
Multivariate				
Main device access site	2.319	1.283	4.192	0.005
Calcification 50% to 75% of the CFA circumference	3.291	1.378	7.858	0.007

Abbreviations: CFA, common femoral artery; CI, confidence interval; Fr, French gauge; IBD, iliac branch device.

**Table 6.** Univariate and Multivariate Binary Regression Analysis for Determinants of Access Complications After Large Sheath Size Punctures.

	Odds ratio	95% CI lower limit	95% CI upper limit	p value
Univariate				
Prior surgical access	0.666	0.234	1.894	0.466
Male gender	1.353	0.654	2.797	0.415
Diabetes mellitus	0.260	0.154	1.657	0.260
Previous stroke	1.223	0.533	2.804	0.635
Peripheral arterial disease	2.280	1.178	4.415	0.014
EVAR	0.422	0.165	1.085	0.073
Increase per Fr sheath size	1.250	1.085	1.440	0.002
Increase in sheath-vessel ratio	0.512	0.250	1.047	0.067
Main device access site	2.520	1.254	5.066	0.010
Procedure time (hours)	1.604	1.314	1.956	<0.001
Multivariate				
Main device access site	2.394	1.071	5.351	0.033
Increase in sheath-vessel ratio	0.328	0.127	0.847	0.021
Procedure time (hours)	1.653	1.339	2.041	<0.001

Abbreviations: CI, confidence interval; EVAR, endovascular aortic repair; Fr, French gauge.

## Discussion

This study analyzes the impact of previous surgical femoral artery exposure or repair on the feasibility and outcomes of percutaneous (complex) EVAR in 632 patients. It demonstrates that previous surgical femoral artery exposure or repair does not result in decreased technical success of the percutaneous access and does not lead to a higher amount of vascular access complications, compared with a primary percutaneous femoral access.

Total percutaneous access has become the most used access strategy in standard and complex EVAR.^11–13^ As a result of an increase in endovascular techniques and indications, patients undergoing (complex) endovascular aortic aneurysm repair have become older with more comorbidities and have undergone more often previous aortic intervention with surgical access of the femoral arteries.^14^ A re-do percutaneous access is often not considered in these patients with a prior surgical femoral artery exposure because of expectation of technical failure.^15^ However, a re-do open surgical femoral access may not be an easy and straightforward approach either and has its own complications, such as bleeding and infection.^16,17^ Therefore, patients with previously surgically exposed or repaired femoral arteries could actually benefit more from a successful percutaneous approach for EVAR.

In our retrospective study, the primary outcome of technical success, defined as absence from surgical conversion, was 95.3% in intact groins and 93.3% in groins with a prior surgical approach, which is comparable with the results in literature. Jaffan et al have published a systematic review analyzing 36 pEVAR studies and reported a mean technical success rate of 94%.^18^ For percutaneous access in complex aortic aneurysms, Timaran et al described a technical success rate of 92% in fenestrated EVAR and de Souza et al reported a technical success rate of 95% during F/B EVAR.^19,20^ It is conspicuous that in our study failure of the percutaneous access in re-do groins occurred mostly in the early stage of the procedure, when performing preclosure or gaining sheath access. Percutaneous access of prior previously surgically exposed or repaired femoral arteries seems more burdensome to achieve, but after that obstacle has been overcome, the final closure could even be safer as only 2 conversions were necessary at the end of the procedure. A reason for the probably easier achievement of hemostasis in re-do groins could be the stiff scar tissue sealing the puncture trajectory if the ProGlide sutures do not accomplish complete closure.

Pecoraro et al reported a percutaneous femoral access using a predilation technique in 16 patients with prior surgical vascular procedures in the groin and 9 patients with prior open femoral access for an endovascular procedure in a case series. They demonstrated a failure in 2 patients: 1 needed surgical repair and in the other case hemostasis had to be achieved with a covered stent. Unfortunately, the authors did not provide any information about either postinterventional complications or the need for surgical revision.^21^

In our univariate analysis, previously reported risk factors for failure of percutaneous femoral access, such as female gender, diabetes mellitus, and previous stroke, were not associated with percutaneous access failure. However, increasing sheath size was close to be a significant risk factor for both conversion to open approach and postinterventional access complications.^13,22,23^ Importantly, previous surgical femoral artery exposure or repair was not associated with technical access failure or access complications. The main device access site was a significant predictor for failure of percutaneous access and postinterventional access complications. This might be an expression of several factors, one of which is that larger sheaths were introduced through the main device access site than the contralateral groin and larger sheath size did show a trend to increase the risk to have an access complication. Furthermore, it has to be considered that the main device access site is manipulated more because of the insertion of several devices and will often have a large sheath inside for a longer time period than the contralateral groin. This consideration is supported by the finding that increase in procedure time was an independent risk factor for postinterventional access complications.

The re-do group contained more active smokers, patients with PAD, arterial hypertension, a higher ASA classification, and more patients who received an IBD procedure. This difference reflects the history and status of the vascular disease of the re-do patients. Furthermore, the accessed femoral artery diameter was larger with a consequently increased sheath-vessel ratio. This trend might be caused by selection bias of the operators to reject relatively small previously surgical exposed femoral arteries for percutaneous access. The larger diameter of the access vessel in the re-do group might have contributed to the equal technical success, as the sheath to femoral artery ratio is a known risk factor for access complications.^10,22,24,25^ Despite the baseline difference in PAD, which is another potential risk factor for percutaneous failure, there were no significant differences in outcome between the re-do and intact groin access groups. Finally, in 68 of the re-do groins, a scar was visible on the preoperative CT scan. Earlier performed studies described the presence of a groin scar and PAD as a risk factor for failure of percutaneous access.^7,10,21,23^ We were not able to confirm the presence of a groin scar as a risk factor for percutaneous access failure.

## Limitations

Shortcomings of our study include the retrospective design, which may limit the ability to collect reliable data on some of the end points and cause a selection bias. The re-do group has a certain heterogeneity because it consists of patients who previously received a limited surgical approach of the femoral artery through oblique incision and patients who underwent extensive surgical femoral artery revascularization. A further limitation could be the heterogeneous group of endovascular aortic interventions, despite the equal distribution over the 2 groups. Finally, this study was performed in 2 high-volume centers with a significant experience in percutaneous femoral access. Therefore, the results of our study may be difficult to be translated to low-volume centers.

## Conclusion

In our cohort, prior open surgical femoral artery exposure or repair, either by limited oblique incision or by extensive longitudinal dissection, did not adversely affect the technical success and clinical outcomes of percutaneous (complex) endovascular aortic aneurysm repair.
